# Safety Evaluation, *in Vitro* and *in Vivo* Antioxidant Activity of the Flavonoid-Rich Extract from *Maydis stigma*

**DOI:** 10.3390/molecules201219835

**Published:** 2015-12-10

**Authors:** Ke-Zheng Peng, Xiudong Yang, Hong-Li Zhou, Shu-Xia Pan

**Affiliations:** 1 College of Chemical and Pharmaceutical Engineering, Jilin Institute of Chemical Technology, Jilin 132022, China; pkz1994.3.1@163.com (K.-Z.P.); yangwt_1981@163.com (X.Y.); 2 College of pharmacy, Zhengzhou University, Zhengzhou 450001, China; 3 Department of Mathematics, School of Public Health, Jilin Medical College, Jilin 132013, China; pan4652405@163.com

**Keywords:** flavonoids, *Maydis stigma*, acute toxicity, antioxidant activity

## Abstract

This study aimed to assess the acute toxicity and safety of flavonoid-rich extract from *Maydis stigma* (FMS) in mice. The *in vitro* antioxidant activity of FMS was determined by 1,1-diphenyl-2-picrylhydrazyl (DPPH) and 2,2′-azinobis-(3-ethyl-benzthiazoline-6-sulphonate) (ABTS) scavenging assays. Furthermore, the *in vivo* antioxidant of FMS against ethanol-induced oxidative damage in mice was determined by analysis of the serum total superoxide dismutase (T-SOD) activity, malondialdehyde (MDA) content, liver tissue glutathione (GSH) content, and protein carbonyl (PC) content in liver tissue. The oral administration of FMS at doses of 30 g/kg did not cause death in mice, and there were no significant biologically adverse effects in mice. These results indicated that the median lethal dose (LD_50_) is higher than this dose. The IC_50_ values of FMS for the DPPH and ABTS scavenging activity were 50.73 and 0.23 mg/mL, respectively. Meanwhile, FMS could significantly enhance T-SOD activity, reduce MDA content in the serum, increase GSH content, and decrease PC content in the liver tissue at the tested doses (25, 50, 100, 200 mg/kg·day). These results indicate that FMS can be generally regarded as safe and used potentially as a bioactive source of natural antioxidants.

## 1. Introduction

Maize is the world’s third most widely grown crop [1], which has not only been used as food and animal feed for thousands of years, but also as industrial material for producing starches, oils, and ethanol [2]. *Maydis stigma* is the stigma of the maize female flower. It was reported to contain various biological active compounds, such as polysaccharides [3], flavonoids [4], steroids, tannins [5], saponins, Ca, K, Mg and Na salts, amino acids, organic acids, *etc.* Based on its chemical composition, *Maydis stigma* has many pharmacological activities, like antipyretic choleretic [6], antioxidant [7,8], antibacterial [9], anti-inflammation [10], anti-depressant [11], anti-fatigue [12], anti-diabetic [13], *etc.* Meanwhile, because of its low price and high medicinal value, it is attracting increasing attention of researchers.

It has been reported that the potential pharmacological activities of *Maydis stigma* are related to the bioactive constituents of the plant such as flavonoids [14]. Flavonoids exist widely in plants, and many studies have indicated that they can effectively scavenge reactive free radicals to protect the cells against free radical oxidation damage [15,16]. For instance, the flavonoids extracted from different plants, such as blueberry [17], calamondin [18], saffron [19], and *Radix scutellariae* [20] were demonstrated to have high antioxidant activity.

Meanwhile, many researches have described that flavonoids of *Maydis stigma* possess a potent antioxidant activity *in vitro* [21,22]. Besides, it has been suggested that flavonoids from *Maydis stigma* inhibit lipid peroxidation, increase anti-oxidant enzyme levels, and improve the exercise tolerance of mice [23]. Therefore, it is important to study the acute toxicity and safety of the purified flavonoids from *M. stigma* as well as their antioxidant activity.

However, there are few reports on the toxicological aspects of the flavonoids extracted from *M. stigma* so far. The acute toxicity test is the most basic method of the testing stage of the toxicology safety assessment, and plays a key role in chemical toxicity assessment [24]. Therefore, further studies on the acute toxicity of flavonoids extracted from *M. stigma* are essential to ensure its safe application.

The aim of this study is to evaluate the acute toxicity, safety, and antioxidant activity of the flavonoids extracted from *M. stigma* (FMS) by the following tests of oral acute toxicity in mice, *in vitro* antioxidant activity, and *in vivo* antioxidant activity in mice.

## 2. Results and Discussion

### 2.1. Acute Toxicity in Mice

#### 2.1.1. General Clinical Signs

During a 14-day observation period, there were no abnormal signs in any of the groups, which included addition of body weight, as well as food and water consumption. Besides, the solutions of FMS at the dose of 30 g/kg did not cause the death of male and female mice over 14 days. The results revealed that FMS is non-toxic. Therefore, the results indicated that the oral lethal dose (LD_50_) value for oral administration of FMS in male and female mice is higher than 30 g/kg.

**Figure 1:**
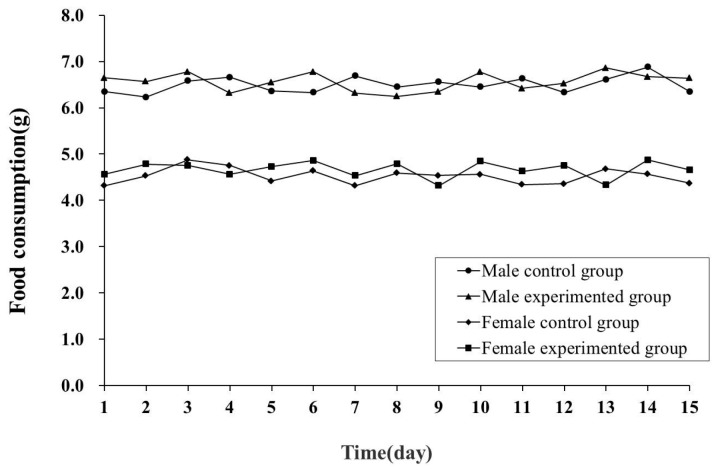
Food consumption of mice in the acute toxicity test.

Table 1 and Figure 1 show the body weights and food consumption of mice in the acute toxicity test, respectively. The body weight gain and the food consumption of the experimented group mice were similar to the control group and there were no significant differences between the two groups (*p* > 0.05). The results showed that, compared to the control group, there were no significant effects of FMS on body weight and food consumption of male and female mice at a dose of 30 g/kg.

**Table 1:** Body weights of mice in the acute toxicity test.

Group	0 day Weight (g)	14 day Weight (g)	Addition of Weight (g)
Male control group	21.95 ± 2.35	25.35 ± 3.04	3.40 ± 1.08
Male experimented group	21.63 ± 1.98	25.27 ± 2.76	3.64 ± 1.56
Female control group	18.05 ± 1.75	21.88 ± 2.09	3.83 ± 0.95
Female experimented group	18.33 ± 2.56	22.13 ± 2.82	3.80 ± 1.12

Values are mean ± SD for 5 mice in each group. Time (day).

The organ coefficient is the ratio of the organ weight to the body weight of the experimental animal, which is commonly used in toxicological evaluation of medicines. With organ damage caused by poisoning, the organ’s weight will change. So the organ coefficient can reflect the pathological changes caused by poisoning [25]. Table 2 showed the organ coefficient of mice in the acute toxicity test. As shown in the table, the organ coefficients of the experimented group mice were similar to the control group and there were no significant differences (*p* > 0.05). In addition, after 14 days, no organs were observed to have obvious lesions in the mice of any of the groups. Therefore, there were no obvious pathological changes in the main organs (heart, liver, spleen, lung, kidney, thymus, brain, bladder, uterus, testis, epididymis, seminal vesicle) of male and female mice at a dose of 30 g/kg.

**Table 2:** Organ coefficient of mice in the acute toxicity test (mg/g).

	Male Control Group	Male Experimented Group	Female Control Group	Female Experimented Group
Heart	9.18 ± 1.57	8.84 ± 1.39	7.55 ± 0.75	7.01 ± 0.98
Liver	92.33 ± 2.62	94.28 ± 3.51	77.15 ± 2.33	78.91 ± 3.06
Spleen	5.15 ± 1.17	5.29 ± 2.01	4.88 ± 0.95	4.60 ± 0.82
Lung	9.72 ± 1.58	9.93 ± 1.35	7.99 ± 1.14	7.91 ± 1.85
Kidney	25.70 ± 2.37	25.46 ± 1.99	17.82 ± 1.13	18.29 ± 1.54
Thymus	2.33 ± 0.75	2.68 ± 0.93	2.01 ± 0.50	2.16 ± 0.37
Brain	10.43 ± 2.54	9.90 ± 1.35	8.57 ± 1.18	8.26 ± 1.05
Bladder	1.25 ± 0.20	1.08 ± 0.32	0.86 ± 0.21	0.77 ± 0.31
Testis (Uterus)	6.22 ± 0.79	6.32 ± 0.63	5.14 ± 1.83	5.57 ± 1.49
Epididymis	2.03 ± 1.24	2.17 ± 1.05		
Seminal vesicle	4.84 ± 1.38	5.03 ± 1.60		

Values are mean ± SD for 5 mice in each group.

#### 2.1.2. Serum Biochemical Parameters

Table 3 presents the serum biochemical parameters of mice in the acute toxicity assay. In general, there were no biologically significant adverse effects. Compared to the control group, FMS at doses of 30 g/kg did not induce significant changes of the biochemical parameters (glucose (GLU), alkaline phosphatase (ALP), alanine aminotransferase (ALT), aspartate aminotransferase (AST).

In summary, the mice in the experimented groups were orally administered with FMS and a dose up to 30 g/kg did not cause any adverse effects in mice when compared to the control group.

**Table 3:** Serum biochemical parameters of mice in the acute toxicity test.

Group	Male Control Group	Male Experimented Group	Female Control Group	Female Experimented Group
ALT (U/L)	49.28 ± 3.52	51.72 ± 4.13	37.91 ± 2.78	35.82 ± 3.03
AST (U/L)	130.03 ± 16.76	125.63 ± 20.27	145.84 ± 25.73	148.01 ± 18.46
ALP (U/L)	118.31 ± 21.54	126.29 ± 18.66	106.51 ± 22.03	102.41 ± 19.14
GLU (mmol/L)	4.83 ± 1.03	4.22 ± 0.98	4.95 ± 1.18	5.12 ± 1.32

Values are mean ± SD for 5 mice in each group.

### 2.2. In vitro Antioxidant Activity

#### 2.2.1. DPPH Radical Scavenging Activity

**Figure 2:**
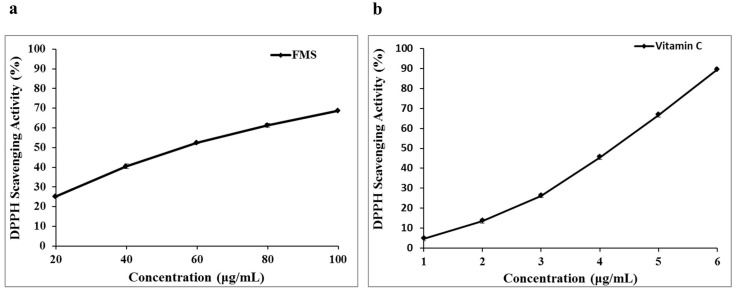
1,1-Diphenyl-2-picrylhydrazyl (DPPH) radical scavenging ability of *Maydis stigma* (FMS) (**a**) and vitamin C (**b**).

Figure 2 presents the DPPH scavenging activity of FMS, with vitamin C used as positive control. FMS and vitamin C were found to scavenge DPPH radical in a concentration-dependent manner. The DPPH radical scavenging ability of FMS was 25.72%–71.19% at concentrations 20.0–100.0 μg/mL, respectively. Meanwhile, the DPPH radical scavenging ability of vitamin C was 4.74%–89.42% at concentrations 1.0–6.0 μg/mL, respectively. IC_50_ values (calculated with the statistical programm SPSS 19.0) were 50.73 ± 0.65 μg/mL and 3.86 ± 0.18 μg/mL for FMS and vitamin C, respectively (*p <* 0.05). The results showed that FMS was demonstrated to have potent scavenging activity against the DPPH radical. However, the DPPH radical scavenging ability of FMS was weaker than that of vitamin C.

#### 2.2.2. ABTS Radical Scavenging Activity

**Figure 3:**
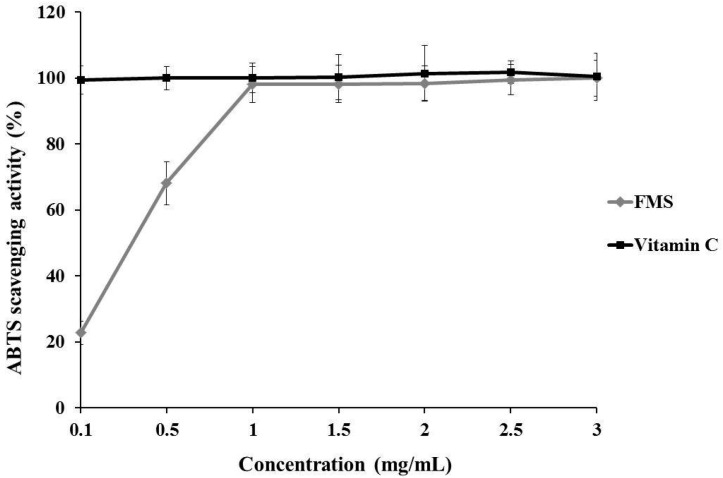
2,2′-Azinobis-(3-ethyl-benzthiazoline-6-sulphonate) (ABTS) radical scavenging ability of FMS and vitamin C.

Figure 3 presents the ABTS radical scavenging ability of FMS and vitamin C. FMS reveals a dose-dependent response at concentrations from 0.1–1.0 mg/mL. At the concentrations of 1.0–3.0 mg/mL, the activities were not significant increased. Also at these concentrations, ABTS radical scavenging ability of FMS ranged from 22.75%–98.03% while the vitamin C ranged from 99.31%–100.0%. Additionally, IC_50_ of FMS was 0.23 ± 0.03 mg/mL (*p <* 0.05). The results showed that FMS has a potent scavenging activity on ABTS radical at concentrations from 0.1–3.0 mg/mL. The ABTS radical scavenging ability of FMS was slightly weaker than vitamin C.

### 2.3. In Vivo Antioxidant Activity in Mice

#### 2.3.1. Effect of FMS on Organ Weights

As shown in Table 4, the organ weights of the model group, positive control group, and dose groups were compared with the control group and there were no significant differences (*p* > 0.05). After the 30 day period, no organs were observed to have obvious lesions in mice of all groups. The results showed that, compared to the control group, there were no obvious pathological changes in the main organs of mice (liver, spleen, lung, kidney) at a dose of 25–200 mg/kg·bw·day.

**Table 4:** Effect of *Maydis stigma* (FMS) on organ weights in mice.

Group	Liver (mg/g)	Spleen (mg/g)	Lung (mg/g)	Kidney (mg/g)
Control	53.88 ± 2.83	4.60 ± 1.14	6.69 ± 0.98	12.81 ± 2.18
Model	55.15 ± 3.59	4.25 ± 1.95	6.07 ± 0.51	13.57 ± 1.51
Dose of 25 mg/kg·bw·day	55.91 ± 2.65	3.91 ± 1.38	6.86 ± 1.03	13.22 ± 1.38
Dose of 50 mg/kg·bw·day	54.06 ± 2.72	4.01 ± 2.20	6.95 ± 0.88	13.25 ± 1.76
Dose of 100 mg/kg·bw·day	51.29 ± 4.04	4.13 ± 1.85	6.34 ± 0.69	12.78 ± 1.58
Dose of 200 mg/kg·bw·day	54.30 ± 2.23	3.72 ± 1.66	6.51 ± 0.72	13.04 ± 1.91
Positive control	52.94 ± 3.28	3.80 ± 2.09	6.30 ± 0.50	13.48 ± 2.37

Values are mean ± SD for 10 mice in each group.

#### 2.3.2. Effect of FMS on the Activity of T-SOD and the Content of MDA in Serum

The lipid peroxidation process, which is triggered by free radicals, generates some final products of polyunsaturated fatty acid peroxidation in cells [26]. Malondialdehyde (MDA) is a frequently used parameter of lipid peroxidation [27]. Superoxide dismutase (SOD) is an essential antioxidant enzyme in the body, which can effectively scavenge the superoxide radical, and protect the body from oxidative stress [28].

Table 5 shows the activity of T-SOD and the content of MDA in the serum. As is shown in the table, the activity of T-SOD in model group is lower than that in control group (*p <* 0.01), and the content of MDA in the model group is higher than that in the control group (*p <* 0.01), which indicated that ethanol could cause oxidative damage to mice, and reduce the antioxidant capacity of mice serum.

**Table 5:** Effect of FSM on the activity of total superoxide dismutase (T-SOD), the content of malondialdehyde (MDA) in serum and the content of antioxidant, glutathione (GSH), protein carbonyl (PC) in liver tissue.

Group	T-SOD (U/mL)	MDA (nmol/mL)	GSH (mg/gprot)	PC (nmol/mgprot)
Control	238.11 ± 30.99 ^a^	7.23 ± 1.17 ^a^	14.25 ± 0.92 ^a^	3.02 ± 1.10 ^a^
Model	134.47 ± 33.38 ^b^	13.41 ± 1.00 ^b^	10.34 ± 1.15 ^b^	5.86 ± 1.05 ^b^
Dose of 25 mg/kg·bw·day	185.97 ± 21.77 ^a^	8.64 ± 1.24 ^c^	13.42 ± 1.33 ^a^	4.22 ± 0.66 ^a^
Dose of 50 mg/kg·bw·day	192.76 ± 17.51 ^a^	6.95 ± 0.97 ^a^	14.34 ± 1.66 ^a^	3.87 ± 0.83 ^a^
Dose of 100 mg/kg·bw·day	200.38 ± 13.30 ^a^	7.26 ± 0.76 ^a^	16.10 ± 2.05 ^a^	2.98 ± 0.66 ^a^
Dose of 200 mg/kg·bw·day	231.53 ± 28.31 ^a^	6.71 ± 0.65 ^a^	18.01 ± 1.41 ^c^	1.44 ± 0.69 ^c^
Positive control	182.58 ± 28.68 ^a^	10.08 ± 0.59 ^d^	16.08 ± 1.14 ^a^	2.36 ± 1.00 ^a^

Values are mean ± SD for 10 mice in each group. Different letters in the same column represent statistically significant differences (*p <* 0.05).

The results indicated that the activity of T-SOD in the model group is significantly lower than that in the dose groups (*p <* 0.01) and positive control group (*p <* 0.01), and the content of MDA in the model group is significant higher than that in the dose groups (*p <* 0.01) and positive control group (*p <* 0.01). Pre-treatment of mice with FMS could significantly protect, in a dose dependent manner, against T-SOD depletion and MDA increment induced by acute EtOH administration. In addition, compared with the model group, pre-treatment with vitamin C was also found to significantly enhance the T-SOD activity and decrease the content of MDA. There are no significant differences of the enhancement of T-SOD activity between the positive control group and FMS dose groups. However, the reduction effects of the MDA content in FMS-treated groups were significantly higher compared to the positive control group. These results show that FMS prevents ethanol-induced T-SOD depletion and MDA increment in a dose-dependent manner.

#### 2.3.3. Effect of FMS on the Content of GSH and PC in Liver Tissue

As an antioxidant, glutathione (GSH) can protect the cell from harmful effects of oxidative stress, and plays a key role in many diseases of aging and pathogenesis, such as cancer, Alzheimer’s disease, Parkinson’s disease, seizure, heart attack, diabetes, *etc.* [29,30]. The content of protein carbonyl (PC) is a widely used parameter to describe the level of oxidative damage to proteins, and reflects the damage in the cell induced by reactive oxygen species [31].

Table 5 lists the comparison of the content of GSH and PC among these groups in liver tissue. As shown in the table, the content of GSH in the model group is lower than that in control group (*p <* 0.01), and the content of PC in the model group is higher than that in control group (*p <* 0.01), which indicates that ethanol does cause oxidative damage to mice, and reduce the antioxidant capacity of mice liver tissue.

Compared with the model group, the content of GSH in the dose groups and positive control group is higher (*p <* 0.01), and the content of PC in the dose groups and positive control group is lower (*p <* 0.01). The results indicate that FMS and vitamin C can significantly reverse the ethanol-diminished GSH content and diminish the protein oxidation induced by ethanol in the liver tissue. In addition, compared with the model group, pre-treatment of FMS at doses of 25, 50, 100, 200 mg/kg·day could significantly increase the GSH content and decrease the PC content in liver tissue in ethanol-treated mice. Meanwhile, the GSH and PC content in livers of mice pre-treated with a dose of 200 mg/kg·bw·day and positive control group were significantly different. This result indicates that FMS at a dose of 200 mg/kg·bw·day has a higher protective effect against ethanol-induced GSH depletion and PC content increment than vitamin C.

In this study, oral administration of FMS at doses of 30 g/kg did not cause the death of male and female mice, and there were no biologically significant adverse effects in mice after an observation time of 14 days. According to the results, we could draw the conclusion that the LD_50_ value for oral administration of FMS in male and female mice is higher than 30 g/kg. In the assays of *in vitro* antioxidant activity, FMS were found to show potent scavenging activity against DPPH and ABTS radical, and IC_50_ values were 50.73 ± 0.65 μg/mL and 0.23 ± 0.03 mg/mL, respectively. However, the scavenging activity on DPPH and ABTS radical of FMS was weaker than vitamin C.

Furthermore, FMS at the tested doses (25, 50, 100, 200 mg/kg·day) can significantly enhance total superoxide dismutase (T-SOD) activity, reduce malondialdehyde (MDA) content in the serum, increase glutathione (GSH) content, and lower protein carbonyl (PC) content in the liver tissue. The results showed that FMS exhibited a potent antioxidant activity *in vivo*, and the antioxidant activity of FMS was significantly better than that of vitamin C.

In recent years, some researches have proved that the extract of *Maydis stigma* possess potent antioxidant activity *in vivo*. It has been reported that the flavone glycoside isolated from the *Maydis stigma* has potent antioxidative and antiapoptotic activities, and it can significantly protect the cell against H_2_O_2_-induced neurotoxicity, which mediates by upregulating the expression of antioxidant enzymes, reduces the accumulation of ROS, and inhibits the damage of DNA [32]. NF-E2-related factor 2 (Nrf2) is a key transcription factor that can regulate expression of antioxidant enzymes and the anti-apoptosis gene. Another study pointed out that the ethanol extract of *Maydis stigma* can protect the cell against oxidative damage induced by radiation, and its antioxidant activity may be related to the up-regulation of Nrf2 [33]. In our study, the antioxidant activity of FMS *in vivo* was significantly better than *in vitro* when compared to vitamin C, which may be caused by the regulation of genes and proteins expression in the cell. Besides, FMS may be metabolized to compounds which possess potent antioxidant activity *in vivo*. However, the mechanism of its antioxidant activity is not yet clear.

## 3. Experimental Section

### 3.1. Chemicals and Materials

*Maydis stigma* was collected from corn fields in October 2014 in Jilin, China. 1,1-diphenyl-2-picrylhydrazyl (DPPH), 2,2′-azinobis-(3-ethyl-benzthiazoline-6-sulphonate) (ABTS), total superoxide dismutase (T-SOD), malondialdehyde (MDA), glutathione (GSH), protein carbonyl (PC), total protein test kits were purchased from Nanjing Jiancheng Bioengineering Institute (Nanjing, China); All other chemicals and reagents were purchased locally and were of analytical grade.

### 3.2. Experimental Animals

Eight weeks old and weight of 25 to 30 g mice, with half male and half female were used for the test of *in vivo* antioxidant activity. Four weeks old and weight of 18 to 22 g mice, with half male and half female were used for the test of acute toxicity. Before the test, mice were reared for 7 days in the experimental environment by feeding common forages. All experimental procedures used in this study were approved by the ethics committee in this institute and all animal experiments were performed in accordance with the ethical standards laid down in the 1964 Declaration of Helsinki. The authors who performed experiments gave their informed consent prior to the study and followed “principles of laboratory animal care” (NIH publication No. 86-23, revised 1985).

### 3.3. Extraction and Determination of Flavonoid Content

The pulverised *Maydis stigma* (300 g) was extracted with water at 80 °C (9 L water, 1 h for the first time, 4.5 L water, 0.5 h for the second time). The extract was filtered through a Whatman No. 1 filter paper to remove the debris and the filtrate was then concentrated to 3 L with a rotary flash evaporator at 40 °C under vacuum (RE-52A, Shanghai Yarong Biochemical Instruments Co. Shanghai, China). Then, the water extract of *M. stigma* was precipitated by the addition of anhydrous ethanol to a final concentration of 70% (*v*/*v*). The mixture was maintained overnight at room temperature. The supernatant was obtained by centrifugation (3500 rpm, 15 min), and then concentrated using a rotary flash evaporator and freeze dried to furnish the flavonoid rich extract from *Maydis stigma* (FMS).

A colorimetric aluminum chloride method was used for determination of the contents of flavonoid [34] with some modification. A dilute solution of FMS in methanol (0.5 mL in 50 mL) was separately mixed with 4.5 mL of methanol and 5.0 mL of 0.01-mol/L aluminum chloride in methanol. The reaction mixture remained at room temperature for 10 min. Then, the absorbance of the reaction mixture was measured at 400 nm using an ultraviolet visible spectrophotometer (UV-2550, Shimadzu Corporation, Kyoto, Japan). The calibration curve was established by preparing rutin solutions at concentrations ranging from 0.005 to 0.125 mg/mL in methanol. The yield of the flavonoids was expressed as mg of rutin equivalents per gram of *M. stigma* on a dry weight basis. The purity of total flavonoids in the extracts of *M. stigma* was 10.45%.

### 3.4. Acute Toxicity in Mice

The test of acute toxicity in mice was investigated by the method of Organization for Economic Cooperation and Development (OECD) Guidelines for Testing of Chemicals [35].

The mice (4 weeks old, weight of 18 to 22 g) were randomly divided into 2 groups, which included the control group and experimented group, also each group had ten mice with half male and half female. Experimented groups were orally administered with solutions which were prepared by dissolving raw extracts of flavonoids from *M. stigma* at a dose of 10 g/kg into the distilled water. Additionally, the control group was orally administered with the equivalent of distilled water. All the mice were orally administered with solutions or distilled water by oral gavage once every 8 h for consecutive 24 h, and a maximum dose up to 30 g/kg body weight. After administration for three times, mice of each group were observed for 14 days for clinical signs and mortality. The clinical signs includes: addition of body weight, food, and water consumption.

On day 14 on completion, all the animals were euthanized and blood samples were collected from mice orbit to separate and centrifuge blood serum under the condition of 5 °C, 2000 r/min for 15 min. All the blood serum was stored at −4 °C for standby. The following biochemical parameters were determined: glucose (GLU), alkaline phosphatase (ALP), alanine aminotransferase (ALT), aspartate aminotransferase (AST). Meanwhile, the main organs (heart, liver, spleen, lung, kidney, thymus, brain, bladder, uterus, testis, epididymis, seminal vesicle) of mice were quickly removed, dried with filter paper and weighed. Finally, the median lethal dose (LD_50_) values were determined according to the test results.

### 3.5. In Vitro Antioxidant Activity

#### 3.5.1. DPPH Radical Scavenging Assay

The antioxidant activity of scavenge DPPH radical species was investigated by the method of Wu *et al.* [36] with some modifications. The extracts of FMS were dissolved in ethanol at different concentrations (20 μg/mL, 40 μg/mL, 60 μg/mL, 80 μg/mL, 100 μg/mL). The 2.0 mL alcoholic solution of DPPH (0.1 mmol/L) was added to the 2.0 mL solution of sample with different concentrations of FMS, respectively. The mixture was reacted at room temperature for 30 min under strict exclusion of light. After that, the absorbance was measured at 517 nm, with ethanol as blank control. Also, vitamin C was used as a reference standard.

DPPH radical scavenging activity was calculated using formula:

RSA (%) = [(A_c_ − A_t_)/A_c_] × 100

where A_c_ and A_t_ is the absorbance of pure DPPH and the absorbance of DPPH in the sample, respectively. The result was expressed as the half maximal inhibitory concentration (IC_50_) of FMS.

#### 3.5.2. ABTS Radical Scavenging Assay

The antioxidant activity of scavenge ABTS radical species was investigated by the method described by Antolovich *et al.* [37] with some modifications. The 5.0 mL ABTS solution (7.0 mmol/L) was mixed with 88 μL potassium persulfate (K_2_S_2_O_8_) solution (140 mmol/L), and the mixture was kept at room temperature for 15 h under strict exclusion of light. After that, the solution was diluted with distilled water to obtain an absorbance of 0.7 ± 0.02 units at 734 nm at a temperature of 30 °C. Then, to perform the ABTS radical scavenging assay, the 3 mL solution of ABTS was added to the 200 μL FMS solution at various concentrations (0.1 mg/mL, 0.5 mg/mL, 1.0 mg/mL, 1.5 mg/mL, 2.0 mg/mL, 2.5 mg/mL, 3.0 mg/mL), respectively. The mixture was reacted at room temperature for 1 h under strictly exclusion of light. After that, the absorbance was measured at 734 nm, with solvent as blank control. Additionally, vitamin C was used as a positive control.

ABTS radical scavenging activity was calculated using formula:

RSA (%) = [(A_c_ − A_t_)/A_c_] × 100

where A_c_ and A_t_ was the absorbance of control and the absorbance of sample or standard, respectively. The result was expressed as the half maximal inhibitory concentration (IC_50_) of FMS.

### 3.6. In Vivo Antioxidant Activity in Mice

All *in vivo* antioxidant activity experiments were conducted according to the Method for the Assessment of Antioxidative Function which was issued by the China Food and Drug Administration. The mice (8 weeks old, weight of 25 to 30 g, female) were randomly divided into 7 groups, which included the control group, model group, positive control group, FMS-treated groups (25, 50, 100, 200 mg/kg·bw·day), and each group had ten mice. Four FMS-treated groups were orally administered with solutions which were prepared by dissolving raw extracts of flavonoids from *M. stigma* with doses of 25, 50, 100, 200 mg/kg·bw·day into the distilled water, respectively. The positive control group was orally administered with solutions by dissolving vitamin C with doses of 200 mg/kg·bw·day into the distilled water. The model group and control group were orally administered with the equivalent amount of distilled water per day.

After 30 days, all the animals were fasted overnight. Then all mice except for the control group were orally perfused 50% ethanol with a dose of 12 mL/kg body weight to make oxidative damage mould [38]. After 6 h, blood samples were collected into a heparinized Eppendorf tube from a mice orbit to separate and centrifuge blood serum under condition of 5 °C, 2000 r/min for 15 min. After that, the mice were killed and the liver, lung, kidney, and spleen of mice were dissected, dried with filter paper, and weighed. All organs were dried with filter paper before weighing. The livers were homogenized in ice-cold 10% PBS (0.02 mol/L, pH 7.2) under centrifugation condition of 5 °C, 2500 r/min for 10 min to collect a clear supernatant liquid. The serum total superoxide dismutase (T-SOD) activity, malondialdehyde (MDA) content, liver tissue glutathione (GSH) content, and protein carbonyl (PC) content in tissue supernatants were chosen as indices to observe the influence of FMS on the antioxidant ability of ethanol oxidation to damage model mice. All the experiments were carried out according to the instructions of commercial kits purchased from the Nanjing Jiancheng Bioengineering Institute (Nanjing, China).

### 3.7. Statistical Analysis

Statistical analysis was conducted using the one-way analysis of variance (ANOVA) with the statistical program SPSS 19.0. The results were expressed as mean ± standard deviation in triplicate. Significant differences were set at *p <* 0.05.

## 4. Conclusions

In conclusion, although the flavonoids extracted from *M. stigma* might have an anti-aging effect because of their potent antioxidant activity, the mechanism of the antioxidant activity is still unclear and whether it is effective for the human body is still unknown. Therefore, additional studies will be necessary to verify the mechanism of antioxidant activity and the toxicological effect on the human body. In view of the potential use of *M. stigma* in the field of the food and drug industry, the flavonoids extracted from *M. stigma* as a natural antioxidant, can be utilized for preventing diseases caused by oxidative stress, and have a broad prospect of application and development.
